# Efficacy and Safety of Collagenase Clostridium Histolyticum in the Treatment of Peyronie's Disease: An Evidence-Based Analysis

**DOI:** 10.3389/fmed.2022.780956

**Published:** 2022-02-18

**Authors:** Dehong Cao, Jinze Li, You Lu, Yin Huang, Bo Chen, Zeyu Chen, Yinzhi Shen, Liangren Liu, Qiang Wei

**Affiliations:** ^1^Department of Urology, Institute of Urology, West China Hospital, Sichuan University, Chengdu, China; ^2^Department of Child Healthcare, West China Second University Hospital, Sichuan University, Chengdu, China; ^3^West China School of Medicine, Sichuan University, Chengdu, China

**Keywords:** Peyronie's disease (PD), Collagenase Clostridium Histolyticum (CCH), sexual function, efficacy, safety, meta-analysis

## Abstract

**Background:**

Peyronie's disease (PD) is a chronic wound healing disorder, mainly involving tunica albuginea. Collagenase Clostridium Histolyticum (CCH) has shown its effectiveness in treating PD, but its efficacy and safety remain controversial, which propelled us to conduct the first evidence-based research on this topic.

**Methods:**

We searched the Web of Science, PubMed, Embase, and ClinicalTrials.gov for related randomized controlled trials (RCTs). A systematic review and meta-analysis were performed to compare the penile curvature deformity (PCD), Peyronie's Disease Questionnaire peyronie's disease symptom bother (PDSB), penile pain score, total treatment-related adverse events (TAEs), and specific adverse events, including penile pain, penile edema, injection site pain, and contusion. Cochrane Collaboration's tool and Review Manager 5.3.0 version were applied, respectively, to evaluate the study quality and heterogeneity.

**Results:**

Four articles (five RCTs) with 1,227 patients were finally included in the meta-analysis. The results revealed that CCH had excellent efficacy in relieving PCD (weighted mean difference [WMD]: −318.77, *p* < 0.001) and PDSB (WMD: −1.20, *p* < 0.001) compared to the placebo group, but there was no difference in the penile pain score (WMD: −0.64, *P* = 0.39) between the two groups. Furthermore, the incidence of TAEs in the CCH group was higher [odds ratio (OR): 12.86, *p* < 0.001].

**Conclusions:**

The current evidence suggests that CCH has a significant effect on treating PD. Considering that all these adverse events are acceptable and curable, CCH could slow the disease progression in the acute phase or act as a substitute for patients unable or unwilling to undergo surgery. However, the conclusion could not be certainly drawn until RCTs with a larger scale proved it.

## Introduction

Peyronie's disease (PD), also known as penile fibrous cavernositis, is a benign chronic disease characterized by the formation of fibroids in the tunica albuginea (1), leading to plaque formation, penile malformation, penile pain, sexual dysfunction, and mental disorders (2). Recent epidemiological surveys have shown that the period of the PD onset is usually 40 to 70 years old, and its prevalence is 3 to 9%. However, due to its low recognition, the prevalence of PD may actually be higher (3–5).

At present, clinical treatment of PD is mainly concentrated on the acute phase of the disease, aiming to prevent disease progression and penile malformation. For patients in the acute phase, non-surgical treatment is the primary choice, and only patients who are in the stable phase or with serious problems are treated with surgery (1, 6). Non-surgical treatments, such as oral drug therapy, intralesional local injection therapy, iontophoresis, and vacuum mechanical traction therapy, have received extensive attention (7–9). However, there is no strong evidence to prove their therapeutic advantages (10).

Local injection therapy for lesions has been accepted recently, because of its rapid drug delivery, high local drug concentration, and ease of clinical operation (7, 11). Currently, drugs for the local injection are mainly CCH, verapamil, and interferon-α2b, of which CCH is the most widely used (9, 12, 13). Previous clinical trials focused on the effectiveness of CCH (14–16), but most of them were reviews or case reports, lacking evidence-based medical evidence, and there are still some clinicians who are concerned about its safety and efficacy. Therefore, we are the first to use meta-analysis to explore the efficacy and safety of CCH in the treatment of PD all over the world.

## Methods

This present study was performed following the Preferred Reporting Items for Systematic Reviews and Meta-Analyses (PRISMA) criteria (17).

### Search Strategy

The following search string terms: “Peyronie's,” “Peyronie's disease,” “penile curvature,” “PD,” “Collagenase Clostridium Histolyticum,” “Xiafle,” “Xiapex,” and “CCH” were used to systematically search the Web of Science, PubMed, Embase, and CinicalTrials.gov date to May 2021 for randomized controlled trials (RCTs) that compared CCH with placebo for PD. The search language was limited to English. In addition, the reference lists of all eligible studies were reviewed manually.

### Study Selection Criteria

If correlative studies suffice to meet all the following criteria, they will be included in this study: (1) RCTs or pseudo-RCTs, (2) Males who were 18 years of age or older, had a regular heterosexual partner, and were clinically diagnosed with PD; (3) These RCTs investigated the effect of CCH in patients with PD and compared it with placebo or blank controls; and (4) The study provided at least one indicator of outcomes that can be analyzed. On the contrary, studies were excluded if: (1) The study data could not be obtained; (2) Studies that combined CCH with other treatments were excluded; (3) Animal experiments, reviews, letters, editorial comments, pediatric articles, case reports, or conference abstracts; and (4) Unpublished articles and non-English articles.

### Data Extraction

After scanning the title, abstract, and full text, two analysts (Cao and Shen) selected the literature in strict accordance with the inclusion criteria and then extracted the data according to the pre-designed table for cross-checking. Any argument on this topic was arbitrated by a third researcher. The extracted data included the first author, year of publication, type of study design, interventions, total number and age of subjects, the follow-up period, and outcome indicators. The following result outcomes were extracted: penile curvature deformity (PCD), Peyronie's Disease Questionnaire Peyronie's disease symptom bother (PDSB), penile pain score, and total treatment-related adverse events (TAEs). Additionally, several common complications, such as penile pain, penile edema, injection site pain, and contusion, were also included.

### Study Quality Assessment

The quality assessment was based on methodological quality assessment criteria recommended by the Cochrane Handbook for Systematic Reviews of Interventions Version 5.1.0 (16). “High risk” stands for the high risk of bias, “low risk” stands for the low risk of bias, and “unclear risk” stands for the absence of adequate information to conduct the bias evaluation. All differences were solved by a third researcher.

### Statistical Analysis

RevMan 5.3.0 (Cochrane Collaboration, Oxford, UK) was used for meta-analysis. Weighted mean difference (WMD) and odds ratio (OR) were used as the effect indexes for continuous and dichotomous data, respectively, and *P* values and 95% CI were given for both. Heterogeneity between studies was judged by Cochran's *Q* and *I*^2^ statistics. When there was a statistical homogeneity between studies (*p* > 0.1 and *I*^2^ < 50%), the fixed effect model was introduced for analysis; otherwise, the random effect model was applied. For all statistical consequences, *p* < 0.05 was considered statistically significant. Sensitivity analysis was performed by excluding one or more studies that led to heterogeneity.

## Results

### Description of Studies

**Figure 1:**
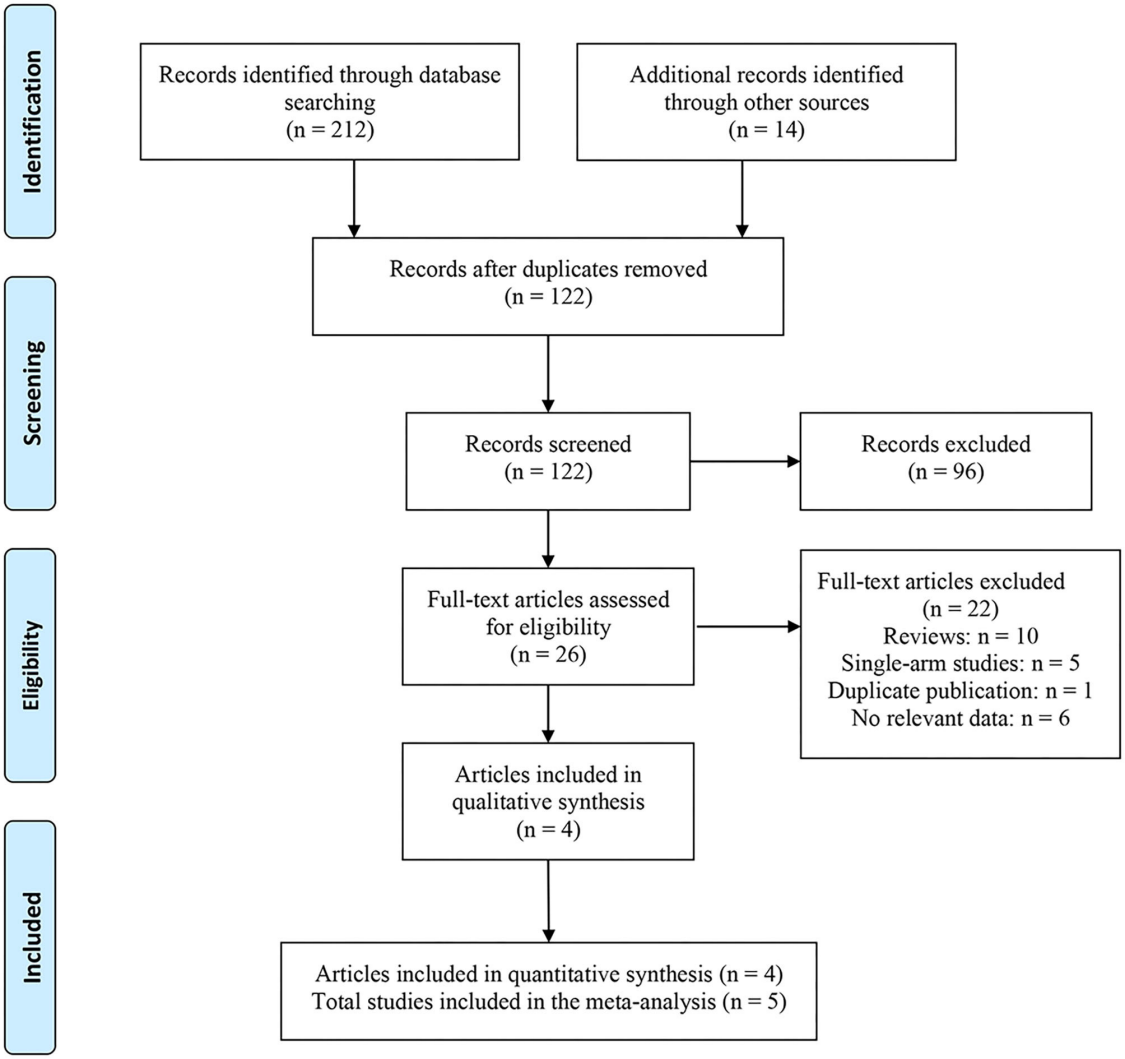
The literature screening process.

**Figure 2:**
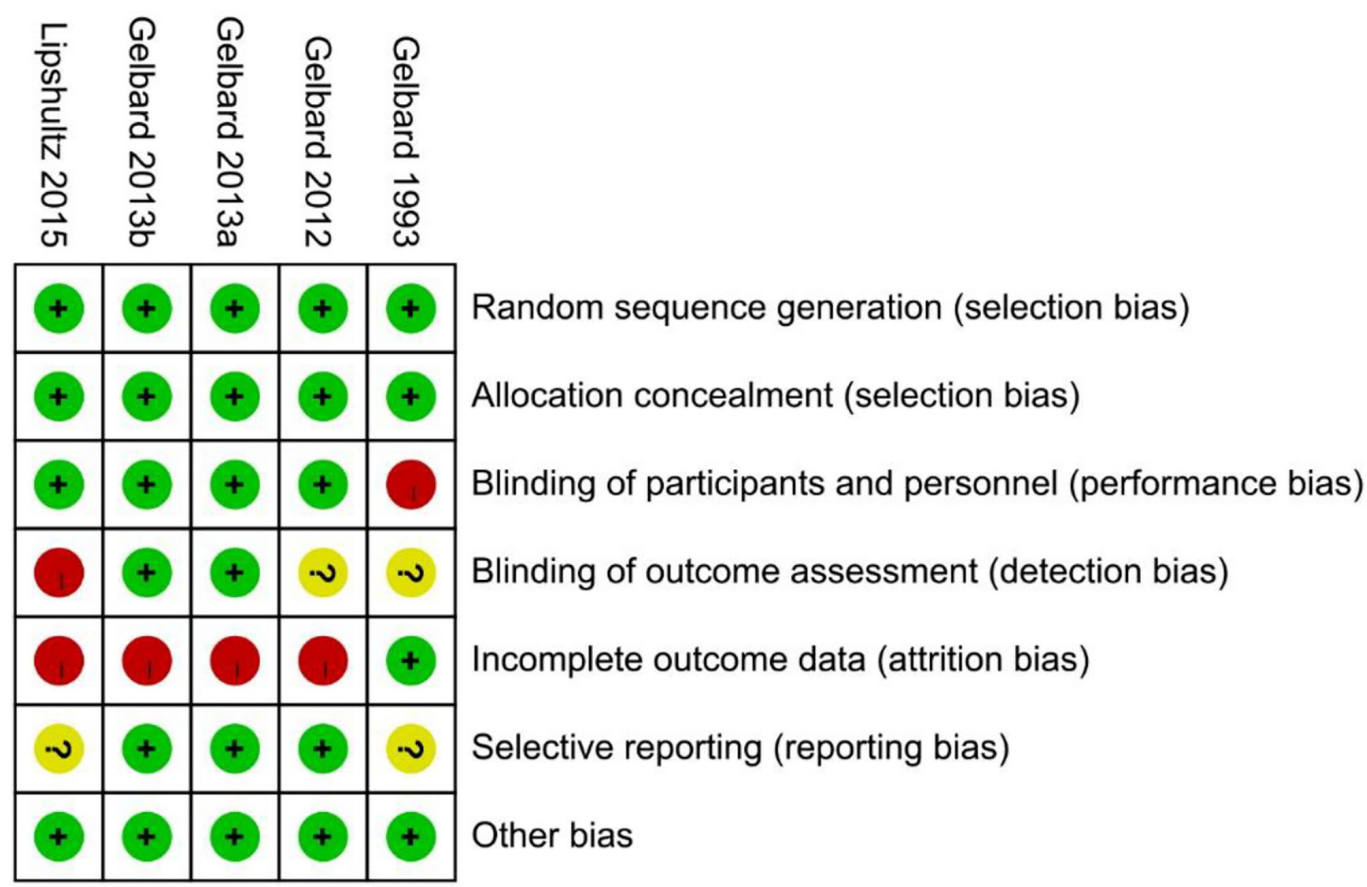
Quality evaluation of included randomized controlled trials (RCTs).

A total of 226 related articles were obtained by a preliminary examination. Two hundred duplicates and unrelated studies were removed, and 26 studies were left. After reading the full text, four articles (five studies) were finally included for meta-analysis (Figure 1) (18–21), and 1,227 patients with PD were involved. Of these, 815 and 412 patients accepted CCH and placebo, respectively. In one of these articles, there were two studies, and they came from different experimental centers, so we named these two studies Gelbard a and Gelbard b (20). In addition, we also looked for partial data available at the Cochrane Central Register of Controlled Trials according to the ClinicalTrials.gov Identifier of the three RCTs. The basic information and baseline characteristics of the incorporated studies are shown in Table 1, and the methodological quality evaluation of RCTs is exposed in Figure 2.

**Table 1:** Basic information and characteristics of studies.

**Authors**	**Year**	**Design**	**LOE**	**CCH/Placebo**		**Intervention**	**Follow-up**	**Outcome measures**
				**Patients (N)**	**Age (years)^a^**			
Gelbard et al. (18)	1993	RCT	1b	22/27	NA	6,000–14,000 unit in 3–7 injections	3 months	TAEs
Gelbard et al. (19)	2012	RCT	1b	111/36	56.9 (7.8)/55.4 (7.0)	1–3 cycles^b^ with a interval of 6 weeks	36 weeks	PCD, PDSB, penile pain score, TAEs, penile pain, penile edema, injection site pain and contusion
Gelbard a (20)	2013	RCT	1b	277/140	57.9 (8.2)/58.2 (8.9)	1–4 cycles^b^ with a interval of 6 weeks	52 weeks	PCD, PDSB, penile pain score, TAEs, penile pain, penile edema, injection site pain and contusion
Gelbard b (20)	2013	RCT	1b	274/141	57.3 (8.8)/57.6 (7.5)	1–4 cycles^b^ with a interval of 6 weeks	52 weeks	PCD, PDSB, penile pain score, TAEs, penile pain, penile edema, injection site pain and contusion
Lipshultz et al. (21)	2015	RCT	1b	131/68	NA	1–4 cycles^b^ with a interval of 6 weeks	52 weeks	PCD, PDSB

*RCT, randomized controlled trial; LOE, Level of evidence; CCH, Collagenase Clostridium Histolyticum; TAEs, treatment-related adverse events; PCD, penile curvature deformity; PDSB, Peyronie's disease questionnaire symptom bother; NA, not available*.

a *Data are presented by median (standard deviation)*.

b *Each treatment cycle consisted of 2 intralesional injections of 0.58 mg CCH or placebo, with an interval of approximately 24–72 h between each injection. Approximately 24–72 h following the second injection of each treatment cycle, subjects in the CCH and placebo groups underwent penile plaque modeling*.

### Penile Curvature Deformity

**Figure 3:**
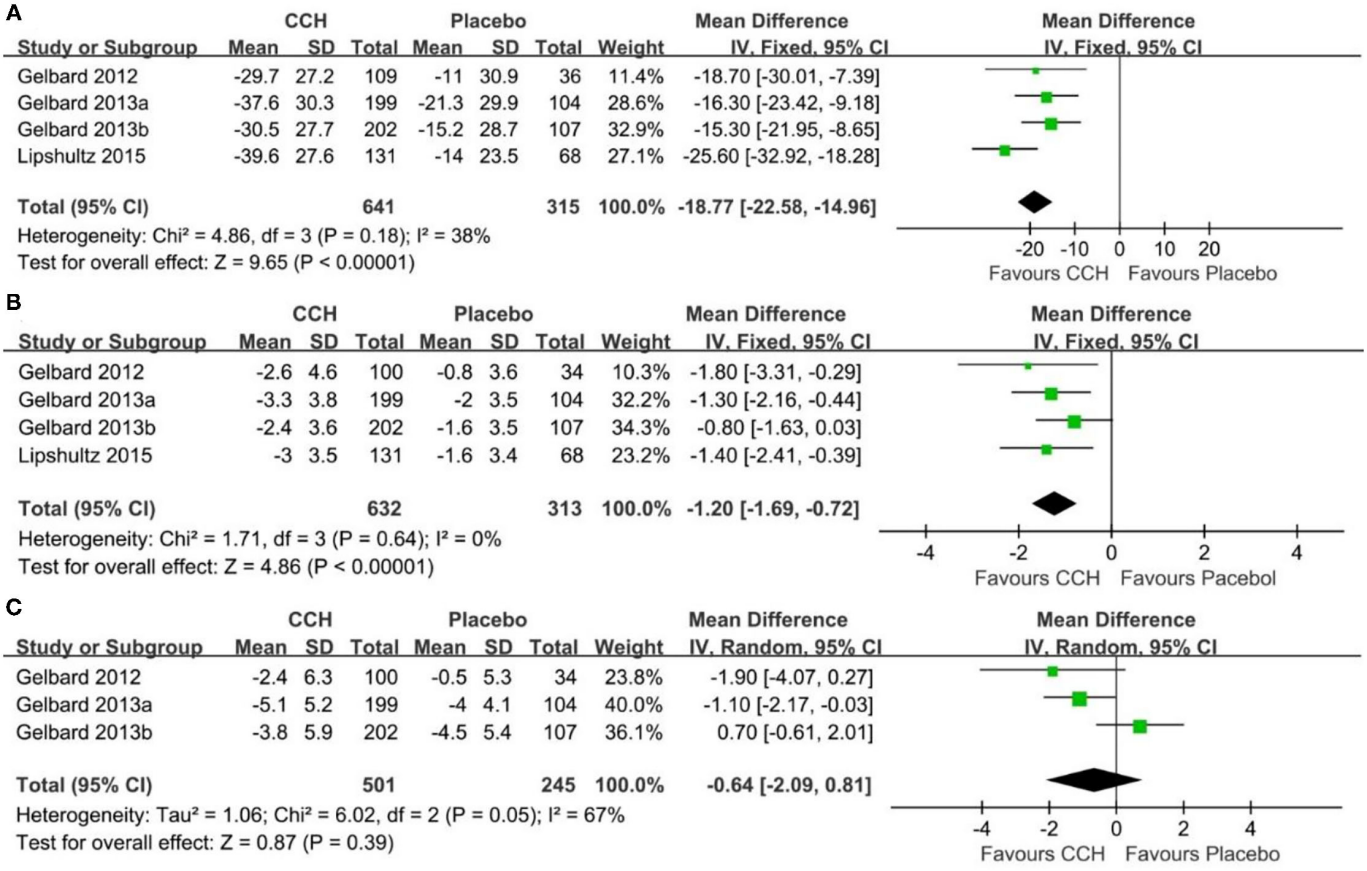
Comparison between Collagenase Clostridium Histolyticum (CCH) and placebo in efficacy. **(A)** Penile curvature deformity, **(B)** Peyronie's disease symptom bothers, **(C)** penile pain scores.

The data of mean percent change in PCD were reported in 4 studies involving 956 patients with PD (18–20). The combined results displayed a significant improvement in the CCH group compared with the placebo group (fixed-effects model; WMD: −318.77; 95% CI: −22.58 to −14.96; *p* < 0.001; *I*^2^ = 38%; Figure 3A).

### Peyronie's Disease Symptom Bother

We extracted the data for this indicator from 4 studies (18–20), which contained a total of 945 patients with PD with 632 patients in the CCH group and 313 patients in the placebo group. Results of the heterogeneity test revealed no significant difference between the two groups (*P* = 0.64; I^2^ = 0%; Figure 3B), and a fixed-effects model was introduced. The comprehensive analysis demonstrated that the PDSB in the CCH group was lower than that in the placebo group (WMD: −1.20; 95% CI: −1.69 to −0.72; *p* < 0.001).

### Penile Pain Score

For the penile pain score, 3 included studies reported this outcome (19, 20). Heterogeneity test results indicated a high heterogeneity (*P* = 0.05, *I*^2^ = 67%) between the CCH group and the placebo group. The combined results showed no significant difference in the penile pain score between the CCH group and the placebo group (WMD: −0.64; 95% CI: −2.09 to 0.81; *P* = 0.39; Figure 3C).

### Adverse Events

**Figure 4:**
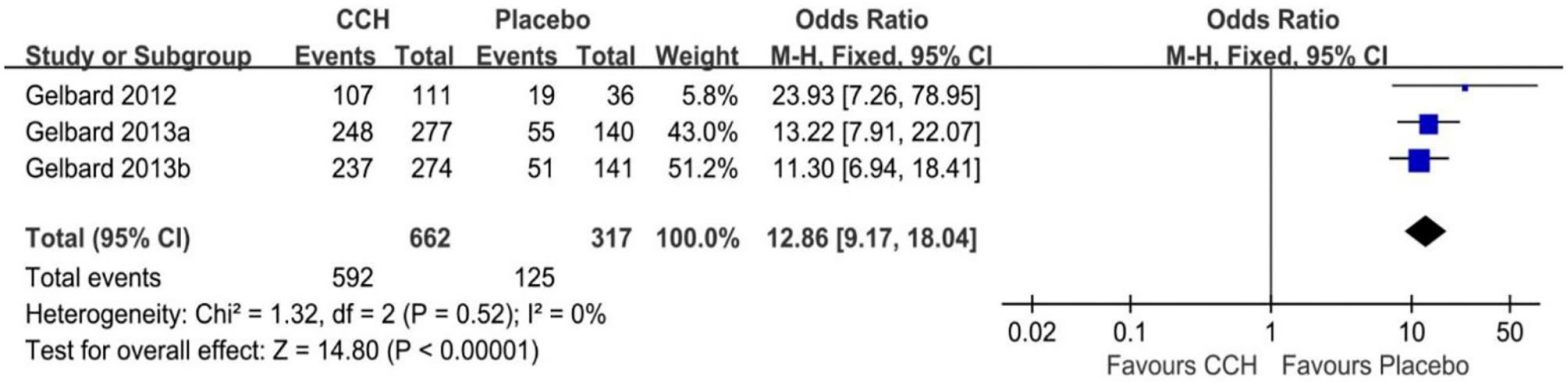
Comparison between CCH and placebo in adverse events.

There were four studies recording the adverse event rate (18–20), and the merged results indicated that the TAEs of the CCH group were significantly higher than that of the placebo group (OR: 12.86; 95% CI: 9.17 to 18.04; *p* < 0.001; *I*^2^ = 0%; Figure 4). In addition, we conducted a meta-analysis of several common complications. In the final statistical analysis, the number of penile pain event in the CCH group was significantly higher, compared with the placebo group (OR: 8.87; 95% CI: 5.43 to 14.50; *p* < 0.001), and others showed similar results (penile edema: OR = 26.86, *p* < 0.001; injection site pain: OR = 7.91, *p* < 0.001; contusion: OR = 14.60, *p* < 0.001; Table 2).

**Table 2:** Comparison of common complications between CCH group and placebo group.

**Outcomes**	**No. of studies**	**No. of patients**	***P* value**	**OR (95% CI)**	**Heterogeneity**			
		**(CCH/Placebo)**			**Chi^**2**^**	**df**	***P***	***I^**2**^* (%)**
Penile pain	4	684/344	<0.001	8.87 [5.43, 14.50]	0.00	2	1.00	0
Penile edema	4	684/344	<0.001	26.86 [6.63, 108.80]	0.82	2	0.66	0
Injection site pain	4	684/344	<0.001	7.91 [4.38, 14.30]	0.38	2	0.83	0
Contusion	4	684/344	<0.001	14.60 [4.13, 51.68]	1.05	2	0.59	0

*CCH, Collagenase Clostridium Histolyticum; OR, odds ratio*.

## Discussion

The incidence of PD increases with the age, and it has been reported that 90% of patients with PD are between 50 and 59 years old (22). As a chronic benign disease, PD could cause erectile dysfunction, which seriously affects the life quality. Unfortunately, there is, currently, no ideal treatment for PD and, thus, made it a hot topic. The CCH, a mixture of AUX-I and AUX-II Clostridium collagenase with high selective hydrolysis activity on Type I and Type III collagen, can directly destroy collagen-based plaques without damaging surrounding elastic tissue and vascular smooth muscle. Gelbard et al. (18) first reported the application of CCH in PD, and they found that the therapeutic effect of CCH was superior to placebo in terms of plaque size and penile deformity. Subsequently, substantial evidence has shown that CCH is an effective non-surgical treatment for PD (13). However, the safety and efficacy of CCH in PD remain controversial. We, therefore, conducted a meta-analysis of the current data to provide a higher level of evidence. As far as we know, our study is the first meta-analysis worldwide that systematically illustrates the efficacy and safety of CCH in the treatment of PD.

The PCD is caused by the formation of fibrous plaques in the tunica albuginea, which may cause great trouble for patients with PD in penetrative intercourse. A clinical trial found that, after two CCH injections, 69 patients had a significant improvement in mean percent change for PCD, and the average reduction in curvature is 23° (23). Evidence synthesis in our meta-analysis also indicated that CCH can significantly reduce PCD in patients with PD compared with placebo (*p* < 0.001), which was consistent with the results of the previous studies (21, 24, 25). Also, the man who is most likely to get PCD improvement is supposed to have curvature between 30° and 60°, longer duration, an international Index for Erectile Function score >17, no calcification, and a set to receive all standard cycles (26). On the other side, however, the practical significance of this improvement remains doubtful and requires further pieces of research, as none of the included studies resulted in a final mean curve <30°, which has been deemed unlikely to inhibit intercourse. Furthermore, for patients with severe curves (>90°), extended curves are recommended to receive surgical management to obtain a superior result (9).

The PD is characterized by penile abnormalities, coital pain, and impaired sexual function, followed by secondary psychological problems. The PDSB and the penile pain score focus on these issues and reflect subjective indicators. Our results confirmed the conclusion from previous studies that CCH can alleviate the PDSB of patients (*p* < 0.001). Increased tissue compliance and positive psychological suggestions for receiving non-surgical therapy could account for this phenomenon (9). In addition, one researcher (27) considered that the reduction of PCD, penile shortening, and pain during sexual intercourse can alleviate PD-related bothers. However, compared with placebo, CCH was not effective in reducing the penile pain score (*P* = 0.39), which may be associated with its side effect, inducing penile pain and bruising (28).

Safety is one of the most important indicators of all doctors' and patients' concerns about CCH treatment. Several studies have examined this topic, but the results were not the same. Tsambarlis et al. believed that patients with PD had a higher incidence of TAEs after receiving CCH, and 80% of the patients expressed a certain degree of dissatisfaction during treatment after therapy (29). On the contrary, several Phase 3 clinical trials have proven that patients treated with CCH have higher TAEs, but they were all acceptable, which were similar to our results. Moreover, the most common complications, such as penile pain, penile edema, injection site pain, and contusion, were slight and could recover without intervention. The occurrence of TAEs is also related to doctors' technical and postoperative care and, therefore, the clinical application should always be treated with caution. It is worth mentioning that corporal rupture, a serious adverse event, must be carefully dealt with to prevent its occurrence, as 34% of the providers encountered at least one case, and 67% of them managed the rupture surgically (30).

This study is the world's first meta-analysis of the efficacy and safety of CCH, and, thus, some limitations could not be avoided. First, although more reliable than the previous form, PDSB and the penile pain score were subjective indicators and could be biased by psychologic status in patients. Second, only 4 studies were included in our study, resulting in inadequate statistical confidence, and 3 of them shared the same first author. However, all studies were randomized controlled trials with a high level of evidence and very low heterogeneity in all results, which could make up for this shortcoming.

## Conclusion

The present meta-analysis demonstrated that CCH had a significant effect on treating PD. This method could relieve bothering symptoms and provide improvement in PCD and sexual function. Its adverse event rate was higher but acceptable. However, more RCTs with a larger sample size are needed to confirm our findings.

## Data Availability Statement

The original contributions presented in the study are included in the article/supplementary material, further inquiries can be directed to the corresponding author/s.

## Author Contributions

LL and QW contributed to conception and design. DC and JL contributed to the acquisition of data and critical revision of the manuscript for important intellectual content. DC, JL, YL, YH, BC, ZC, and YS contributed to the analysis and interpretation of data. DC and JL contributed to the drafting of the manuscript. QW contributed to supervision. All authors read and agreed to the published version of the manuscript.

## Funding

This study was funded by the National Natural Science Foundation of China (Grant No. 82000721), Post-Doctor Research Project, West China Hospital, Sichuan University (Grant No. 2019HXBH089), Health Commission of Sichuan province (Grant No. 20PJ036), and Programs from the Department of Science and Technology of Sichuan Province (Grant No. 2020YJ0054).

## Conflict of Interest

The authors declare that the research was conducted in the absence of any commercial or financial relationships that could be construed as a potential conflict of interest.

## Publisher's Note

All claims expressed in this article are solely those of the authors and do not necessarily represent those of their affiliated organizations, or those of the publisher, the editors and the reviewers. Any product that may be evaluated in this article, or claim that may be made by its manufacturer, is not guaranteed or endorsed by the publisher.
